# Abacavir, zidovudine, or stavudine as paediatric tablets for African HIV-infected children (CHAPAS-3): an open-label, parallel-group, randomised controlled trial

**DOI:** 10.1016/S1473-3099(15)00319-9

**Published:** 2016-02

**Authors:** Veronica Mulenga, Victor Musiime, Adeodata Kekitiinwa, Adrian D Cook, George Abongomera, Julia Kenny, Chisala Chabala, Grace Mirembe, Alice Asiimwe, Ellen Owen-Powell, David Burger, Helen McIlleron, Nigel Klein, Chifumbe Chintu, Margaret J Thomason, Cissy Kityo, A Sarah Walker, Diana M Gibb, CHAPAS-3 trial team

**Affiliations:** a Department of Paediatrics, University Teaching Hospital, Lusaka, Zambia; b Joint Clinical Research Centre, Kampala, Uganda; c Baylor-Uganda, Mulago, Uganda; d Medical Research Council Clinical Trials Unit at University College London, London, UK; e Joint Clinical Research Centre, Gulu, Uganda; f Institute of Child Health, University College London, London, UK; g Department of Pharmacy, Radboud University Nijmegen Medical Centre, Nijmegen, Netherlands; h Division of Clinical Pharmacology, Department of Medicine, University of Cape Town, Cape Town, South Africa

## Abstract

**Background:**

WHO 2013 guidelines recommend universal treatment for HIV-infected children younger than 5 years. No paediatric trials have compared nucleoside reverse-transcriptase inhibitors (NRTIs) in first-line antiretroviral therapy (ART) in Africa, where most HIV-infected children live. We aimed to compare stavudine, zidovudine, or abacavir as dual or triple fixed-dose-combination paediatric tablets with lamivudine and nevirapine or efavirenz.

**Methods:**

In this open-label, parallel-group, randomised trial (CHAPAS-3), we enrolled children from one centre in Zambia and three in Uganda who were previously untreated (ART naive) or on stavudine for more than 2 years with viral load less than 50 copies per mL (ART experienced). Computer-generated randomisation tables were incorporated securely within the database. The primary endpoint was grade 2–4 clinical or grade 3/4 laboratory adverse events. Analysis was intention to treat. This trial is registered with the ISRCTN Registry number, 69078957.

**Findings:**

Between Nov 8, 2010, and Dec 28, 2011, 480 children were randomised: 156 to stavudine, 159 to zidovudine, and 165 to abacavir. After two were excluded due to randomisation error, 156 children were analysed in the stavudine group, 158 in the zidovudine group, and 164 in the abacavir group, and followed for median 2·3 years (5% lost to follow-up). 365 (76%) were ART naive (median age 2·6 years *vs* 6·2 years in ART experienced). 917 grade 2–4 clinical or grade 3/4 laboratory adverse events (835 clinical [634 grade 2]; 40 laboratory) occurred in 104 (67%) children on stavudine, 103 (65%) on zidovudine, and 105 (64%), on abacavir (p=0·63; zidovudine *vs* stavudine: hazard ratio [HR] 0·99 [95% CI 0·75–1·29]; abacavir *vs* stavudine: HR 0·88 [0·67–1·15]). At 48 weeks, 98 (85%), 81 (80%) and 95 (81%) ART-naive children in the stavudine, zidovudine, and abacavir groups, respectively, had viral load less than 400 copies per mL (p=0·58); most ART-experienced children maintained suppression (p=1·00).

**Interpretation:**

All NRTIs had low toxicity and good clinical, immunological, and virological responses. Clinical and subclinical lipodystrophy was not noted in those younger than 5 years and anaemia was no more frequent with zidovudine than with the other drugs. Absence of hypersensitivity reactions, superior resistance profile and once-daily dosing favours abacavir for African children, supporting WHO 2013 guidelines.

**Funding:**

European Developing Countries Clinical Trials Partnership.

## Introduction

In 2014, 91% of 3·2 million HIV-infected children lived in sub-Saharan Africa, but less than 25% of those needing antiretroviral therapy (ART) were receiving it.^1^ Low-cost, scored, dispersible fixed-dose combination (FDC) paediatric tablets of stavudine plus lamivudine plus nevirapine in child-appropriate drug ratios^2^ drove initial ART roll-out to African children, replacing separate syrups, which are costly for programmes and difficult for carers to transport and administer.^3^ However, stavudine was discouraged in 2010^4^ and 2013^5^ WHO guidelines because of high lipodystrophy rates in adults and adolescents. In children, stavudine-associated toxicity has mainly been noted with higher doses than those recommended by WHO and in older children.^6^, ^7^, ^8^

Alternative nucleoside reverse-transcriptase inhibitors (NRTIs) for children younger than 12 years are abacavir or zidovudine. Tenofovir is not licensed for those younger than 2 years and is not recommended by WHO^5^ in those younger than 10 years, primarily because of concerns regarding long-term effects on bone metabolism and renal function in growing children,^9^ although more data are needed. Zidovudine is associated with anaemia, which is of particular concern in malnourished children in endemic malaria areas where underlying anaemia is prevalent. Abacavir is associated with hypersensitivity reactions, although these are rare in Africa^10^ because of a lower risk-allele prevalence.^11^ However, two South African cohorts recently reported lower virological suppression with abacavir than with stavudine,^12^, ^13^ and abacavir is also the most costly NRTI.^14^ Therefore, whether stavudine, given at the WHO recommended doses, should remain an option for young children was unclear.

**Research in context**

**Evidence before this study**

We searched PubMed up to April 27, 2015, using the keywords “HIV”, “child*”, (“stavudine” or “zidovudine” or “abacavir”), not “prevent*” (to exclude a large number of studies looking at zidovudine to prevent mother-to-child HIV transmission), dated after Jan 1, 1996, (when combination ART was introduced). The most relevant nucleoside reverse-transcriptase inhibitors (NRTIs) for treating HIV-infected children when the study started were abacavir, zidovudine, and stavudine; didanosine and tenofovir were not used because of toxicity (genuine or a potential concern, respectively). The WHO conducts systematic reviews as part of guideline development. No existing systematic reviews of randomised controlled trials comparing these NRTIs head-to-head in HIV-infected children were identified in 2010 or 2013, with only one randomised trial directly comparing abacavir and zidovudine in 128 European children, which identfied that abacavir was virologically superior to zidovudine over 5 years follow-up. Recommendations for preferential ordering of zidovudine, abacavir, then stavudine in 2010, and abacavir, zidovudine, then stavudine in 2013, were therefore based primarily on expert opinion balancing toxicity (estimated from observational studies and randomised trials not containing head-to-head comparisons), cost (greater with abacavir), and practicality (particularly availability as part of fixed-dose-combination tablets and once-daily dosing); and, in 2013, also evidence on accumulation of different resistance mutations with sequential use.

**Added value of the study**

This is the first randomised controlled trial in African children, conducting a head-to-head comparison of the three most relevant NRTIs for paediatric treatment, coformulated in NNRTI/NRTI generic fixed-dose-combination paediatric tablets and dosed with WHO drug ratios and weight bands. We identified no major differences between the NRTIs in adverse events, toxicity, clinical, immunological, or viral load endpoints, but did find higher drug susceptibility to relevant second-line NRTIs if abacavir was used first-line, thus providing evidence to support the WHO 2013 recommendation for its use as the preferred first-line NRTI for children. Use of abacavir also enables a once-daily ART regimen to be constructed for children, in line with adults.

**Implications of the available evidence**

Excellent outcomes were obtained on all regimens, showing the importance of widening treatment access for HIV-infected children worldwide. Efforts need to be made to provide abacavir-based combinations where this is possible; but there is no need to move children who are stable on zidovudine-based regimens to abacavir. Further research should investigate the potential for once-daily triple abacavir-based fixed-dose combinations with efavirenz or dolutegravir to further simplify and improve durability of first-line ART for children who will need treatment for much longer than adults.

Since 2003, changes in NRTIs recommended by WHO for children, followed by changes in national guidelines and clinical practice, have occurred with little evidence and no new randomised trials. Therefore, in 2010, when most African children were receiving stavudine-based ART, we aimed to compare stavudine, zidovudine, or abacavir fixed-dose combinations for first-line ART.

## Methods

### Study design and participants

In this open-label, parallel-group, randomised controlled trial (CHAPAS-3), we enrolled confirmed HIV-infected children from Zambia and Uganda—centres were from Zambia—the University Teaching Hospital (UTH), Lusaka; and from Uganda Baylor-Uganda Centre of Excellence, Kampala, and Joint Clinical Research Centre (JCRC), Kampala and Gulu (satellite site)—aged 1 month to 13 years if they were either previously untreated and met WHO 2010^4^ criteria for ART (ART naive; <5 years in Uganda), or on stavudine-containing first-line (non-nucleoside reverse-transcriptase inhibitors [NNRTI]-containing) ART for 2 years or more with screening viral load less than 50 copies per mL and stable CD4 and/or CD4 cell % (ART-experienced; no signs of lipodystrophy; see appendix p 2 for additional eligibility criteria). All children were already on or initiated co-trimoxazole prophylaxis at enrolment (or dapsone if unable to take co-trimoxazole). Caregivers gave written consent; older children aware of their HIV status also gave assent or consent following national guidelines. The trial was approved by Research Ethics Committees in Zambia, Uganda, and the UK.

### Randomisation and masking

Children were randomly assigned (1:1:1) to receive open-label stavudine, zidovudine, or abacavir, together with lamivudine and either nevirapine or efavirenz (at treating paediatrician's discretion: all <3 years received nevirapine). Randomisation was stratified by age (younger than 5 years *vs* 5 years or older), previous ART (naive *vs* experienced), NNRTI (nevirapine *vs* efavirenz), and clinical centre. A computer-generated sequential randomisation list, using the urn probability method^15^ was prepared by the trial statistician and incorporated securely into the trial database at each centre. The list was concealed until allocation, which occurred after eligibility was confirmed by local centre staff, who then did the randomisation.

### Procedures

Scored dispersible fixed-dose combinations of abacavir plus lamivudine, zidovudine plus lamivudine, zidovudine plus lamivudine plus nevirapine, stavudine plus lamivudine, and stavudine plus lamivudine plus nevirapine as so-called baby and junior tablets (Cipla Pharmaceuticals, Mumbai, India) were prescribed following WHO weight bands^5^ (stavudine at lower doses than previous studies^6^, ^7^, ^8^ at 2–4 mg/kg [<10 kg] and at 1·4–2·4 mg/kg [>10 kg] daily). Efavirenz (600 mg double-scored, allowing daily doses of 200 mg, 300 mg, 400 mg, 500 mg, and 600 mg) and nevirapine (200 mg scored) were also supplied for children taking dual NRTI fixed-dose combinations.

Children exited the trial from Oct 30, 2013, to Jan 23, 2014, after a minimum of 96 weeks follow-up. At nurse (6-weekly) and doctor (12-weekly) visits, children were examined, medical history was recorded, adherence was assessed (self-report), and ART was dispensed. At weeks 6, 12, 24, and then 24-weekly, five skinfold thicknesses (triceps, biceps, sub-scapular, supra-iliac, and mid-thigh) and five body circumferences (waist, hip, mid-thigh, mid-upper-arm [MUAC], and torso) were measured to assess lipodystrophy (mean of three measurements); haematology, biochemistry, and CD4 tests were done (results available to clinicians); and plasma was stored for retrospective viral load and resistance testing (results not available to clinicians in real time). Substitutions for toxicity and switches to second-line for failure were at the treating physician's discretion, following WHO guidelines.^5^

### Outcomes

The primary outcome was grade 2 or greater clinical adverse events, confirmed grade 3 laboratory adverse events, or any grade 4 laboratory adverse events^16^ (neutrophils^17^). Clinical primary endpoints were adjudicated against protocol-defined criteria by an endpoint review committee (ERC), masked to allocation, and were also adjudicated for relation to antiretroviral drugs, without knowing the specific ART received. Secondary toxicity outcomes were specific subsets of the primary endpoints (anaemia, neutropenia, lipodystrophy or lipoatrophy, hypersensitivity [also including grade 1 events]), serious adverse events, ART-modifying toxicity (any grade), grade 3/4 adverse events possibly, probably, or definitely related to zidovudine or abacavir or stavudine, and changes in skinfold-thicknesses-for-age and body-circumference-for-age. Secondary efficacy outcomes were viral load suppression, clinical disease progression, change in weight-for-age, height-for-age, and CD4 and ART adherence. Laboratory measures, including viral load, were assayed blind to randomisation. HIV-1 viral load was assayed with the Roche COBAS Ampliprep/Taqman version 2.0 in both Uganda (Joint Clinical Research Centre [JCRC]) and Zambia (Centre for Infectious Disease Research in Zambia [CIDRZ]). Because of small stored sample volumes, most samples were run with a 1/5 dilution with Basematrix 53, giving a lower limit of detection of 100 copies per mL. Drug resistance genotyping was done with either in-house primers (JCRC) or primers from Inqaba Biotec (CIDRZ), with both laboratories using an automated ABI 3730xl sequencer.

### Statistical analysis

Recruiting 470 children gave 85% power to detect a reduction from 20% to 10% in the cumulative incidence of the primary endpoint across the three randomised groups (two-sided α=0·05; appendix p 2). Interim data were reviewed by an independent data monitoring committee (two meetings, approximately annually) using the Haybittle-Peto criterion (p<0·001). Randomised groups were compared with intention-to-treat analysis with log-rank tests for time-to-event outcomes, exact tests for binary outcomes, and generalised estimating equations with independent working correlation for global tests of repeated measures. Analyses were stratified by age group, naive or experienced, and NNRTI, but not by clinical centre because this was not expected to affect outcome (randomisation was stratified by centre for practical reasons; see appendix p 3 for more detail). Data were analysed with Stata version 13.1.

This trial is registered with the ISRCTN Registry number, 69078957.

### Role of the funding source

The funder of the study had no role in the study design, data collection, data analysis, data interpretation, or writing of the report. The corresponding author had full access to all the data in the study and had final responsibility for the decision to submit for publication.

## Results

Between Nov 8, 2010, and Dec 28, 2011, 480 children were randomly assigned: 156 to stavudine, 159 to zidovudine, and 165 to abacavir. After two were excluded due to randomisation error, 156 children were analysed in the stavudine group, 158 in the zidovudine group, and 164 in the abacavir group. More children were ART naive (365 [76%]) than ART experienced (113 [24%]); more were younger than 5 years (337 [71%]); and consequently more received nevirapine (353 [74%]) than efavirenz (more similar percentages >3 years received nevirapine (155 [57%]) and efavirenz (116 [43%]; table 1).

Baseline characteristics were well balanced between randomised groups (table 1). ART-naive children were substantially younger than ART-experienced children (median 2·6 years [IQR 1·6–4·0] *vs* 6·2 years [5·5–7·2], with lower CD4% (median 20% [IQR 13–25] *vs* 35% [30–39]). Median retrospectively assayed viral load was 270 670 copies per mL in ART-naive children (79% >100 000 copies per mL), with three (1%) confirmed less than 100 copies per mL at both screening and enrolment (carers reported no previous ART, no previous samples available). ART-experienced children (all <50 copies per mL) had taken stavudine-based ART for median 3·5 years (IQR 2·6–4·2). The mother or child had received nevirapine or NRTIs for prevention of mother-to-child transmission in 56 (15%) ART-naive and nine (8%) ART-experienced children (table 1).

Median follow-up was 2·3 years among children completing the study (range 1·8–3·1; total 1057 child-years). 25 (5%) children were lost (last seen before October, 2013), including eight (2%) who withdrew consent. 8967 (98%) of 9143 scheduled nurse visits were completed.

**Figure 1:**
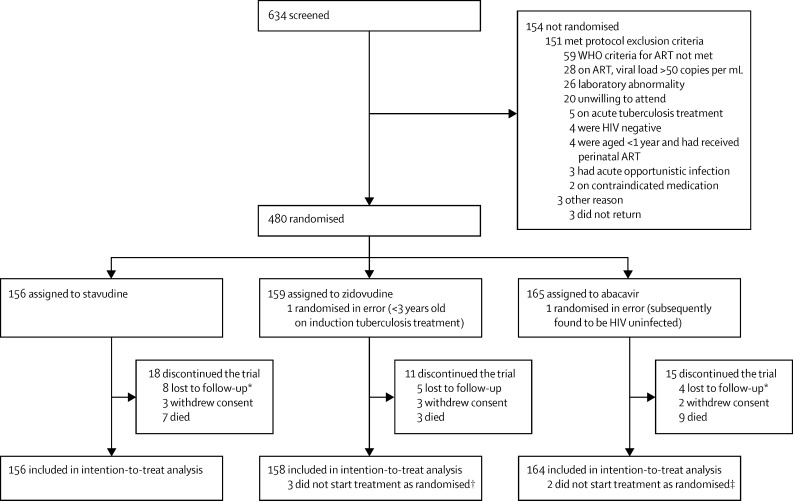
Trial profile ART=antiretroviral treatment. *Includes one not seen after randomisation. †One participant started stavudine and substituted zidovudine at 12 weeks, two started abacavir and did not change (both prescribing errors). ‡Two started zidovudine and did not change (one prescribing error and one child changed regimen to match twin sibling).

Initial ART followed randomisation for 473 (99%) children (figure 1). 445 (93%) remained on their initial treatment throughout follow-up. 33 first-line ART changes occurred among 30 (6%) children: ten (6%) allocated stavudine, 16 (10%) allocated zidovudine, and four (2%) allocated abacavir (p=0·02). Nine changes (three stavudine, four zidovudine, and two abacavir) were nevirapine substitutions for rifampicin-based tuberculosis co-treatment, 14 were nevirapine or NRTI toxicity substitutions, and ten were mostly dispensing errors. Five children ([1%]; all ART-naive, one [1%] stavudine, two [1%] zidovudine, two [1%] abacavir; p=1·0) switched to second-line ART (two clinical, three immunological or virological failure). There was no evidence that self-reported adherence (proportion reporting missing ART doses in the last 4 weeks) across visits through 96 weeks differed between randomised groups (p=0·82).

**Figure 2:**
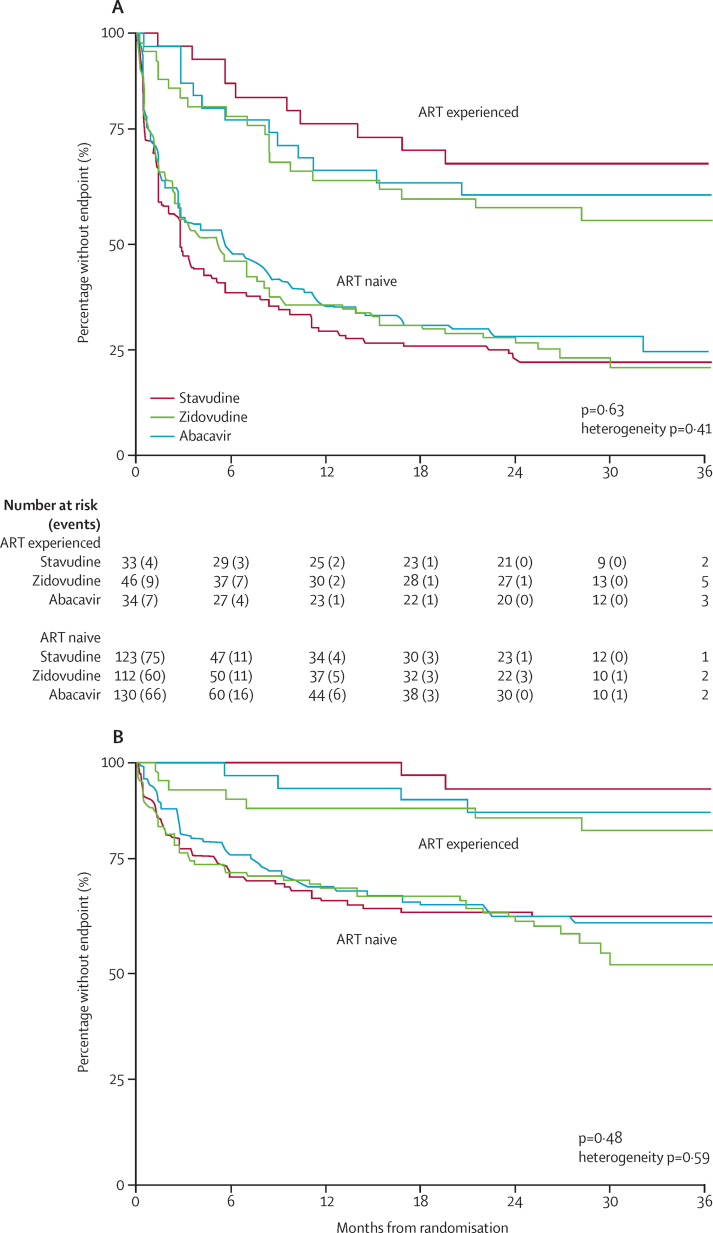
Primary endpoint (clinical adverse event grade 2 or higher, confirmed laboratory grade 3 adverse event, or any laboratory grade 4 adverse event; A) and grade 3 or 4 primary endpoint (B) ART=antiretroviral treatment.

917 grade 2–4 clinical or grade 3/4 laboratory adverse events (877 clinical; 40 laboratory) occurred in 312 children (104 [67%] children allocated stavudine, 103 [65%] children allocated zidovudine, and 105 [64%] children allocated abacavir; p=0·63; figure 2, table 2; appendix p 7). Events were more common in younger ART-naive children than in ART-experienced children (figure 2A), but there was no evidence of heterogeneity in differences between randomised groups (p=0·41). 634 clinical events were grade 2 (481 non-serious respiratory tract infections); excluding grade 2 events gave similar results (p=0·48; figure 2B). 199 serious adverse events occurred in 132 (28%) children, with no difference between randomised groups (p=0·46; table 2). Six (4%) children allocated stavudine, 12 (8%) allocated zidovudine, and five (3%) allocated abacavir had grade 3/4 adverse events judged by the ERC (masked to randomisation) to have at least a possible relation to one of the randomised NRTIs (p=0·10; table 2). No grade 3/4 adverse events or serious adverse events were judged definitely or probably related to stavudine, zidovudine, or abacavir.

14 (3%) children modified ART for toxicity; with significantly more in the zidovudine group (p=0·03; table 2) where eight children substituted zidovudine with stavudine or abacavir for anaemia (n=4), neutropenia (n=3), or leucopenia (n=1). However, there was no evidence of differences between groups in grade 3/4 anaemia (p=0·42 overall; pairwise p>0·25), although more grade 3/4 neutropenia occurred in the zidovudine group (p=0·04 overall; zidovudine *vs* stavudine p=0·03, zidovudine *vs* abacavir p=0·06, stavudine *vs* abacavir p=0·79). Three children substituted ART (all nevirapine to lopinavir plus ritonavir) for hypersensitivity reactions (one grade 2, one grade 3, one grade 4 [Stevens-Johnson; recovered]; none on abacavir). Masked to NRTI received, the ERC adjudicated five stavudine, one zidovudine, and two abacavir primary endpoints as grade 2–4 hypersensitivity reactions (p=0·21; table 2, supplementary tables 1 and 2; appendix p 7); however, neither child on abacavir stopped the drug with no adverse consequences. One additional grade 1 hypersensitivity reaction was reported in the abacavir group; this child also continued abacavir without adverse effects.

Two ART-experienced children substituted stavudine with abacavir after developing facial lipoatrophy (grade 2 [boy, age 6 years, 2·5 years on stavudine]; grade 3 [boy, age 8 years, 5 years on stavudine]). Body circumference increased with time at all measured sites, as expected, while the five skinfold thicknesses decreased similarly in ART-naive and ART-experienced children (appendix p 12, 19), with few differences between randomised groups (appendix p 5). There was no evidence that randomised groups differed in body circumference or skinfold thickness ratios or the sum of the four skinfolds (p>0·1; table 3), or in changes in total cholesterol, LDL, HDL, or triglycerides (p>0·4; appendix p 14).

Disease progression was rare and similar across randomised groups (p>0·3; table 2). All 19 deaths, and 12 of the 14 WHO stage 3 or 4 events, occurred in ART-naive children (seven pneumonia, three tuberculosis WHO stage 3/4 events). Nine of 19 deaths and five of 14 WHO 3/4 events occurred less than 12 weeks after ART initiation, related to pre-enrolment disease severity. There was very little evidence of drug-related mortality (appendix p 15). Change in weight-for-age, height-for-age, or body-mass index-for-age to 96 weeks did not differ significantly between groups (p>0·2).

**Figure 3:**
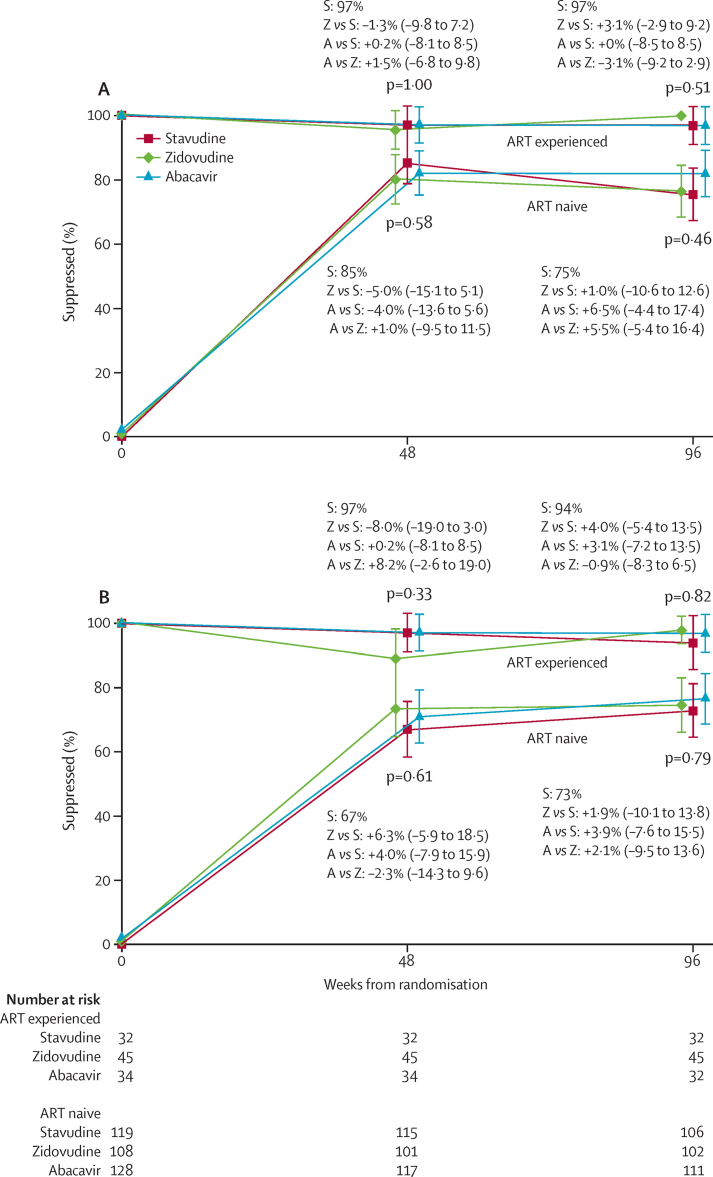
Viral suppression in patients with less than 400 copies per mL (A) and viral less than 100 copies per mL (B) Data are the absolute (95% CI) between-group differences in overall suppression. ART=antiretroviral treatment. S=stavudine. Z=zidovudine. A=abacavir.

Most ART-naive children achieved viral load less than 400 copies per mL by 48 weeks (figure 3A), with no differences between randomised groups (p=0·58; figure 3; appendix p 16). Viral load less than 400 copies per mL was maintained at 48 weeks by more than 96% ART-experienced children (p=1·0). Results were similar between groups at 96 weeks in ART-naive and ART-experienced children (p>0·4), as was viral load suppression less than 100 copies per mL at 48 weeks and 96 weeks (figure 3B). Among ART-naive children, 48-week suppression was better in those with viral load less than 100 000 copies per mL at enrolment (66 [93%] of 71 *vs* 202 [80%] of 254), consistently across randomised groups with no evidence that any NRTI had superior performance in these strata (p_interaction_=0·85). 48-week suppression was similar in ART-naive children aged 3 years or older at enrolment receiving nevirapine (42 [89%] of 47) and efavirenz (88 [91%] of 97), also with no evidence of variation across randomised groups (p_interaction_=0·25). There was also no evidence that 48-week viral load suppression in ART-naive children older than 1 year varied by previous prevention of mother-to-child transmission exposure to nevirapine without NRTI cover (34 [79%] of 43) versus those who had not (219 [85%] of 258; p_interaction_=0·09). There was no evidence of differential CD4% recovery across randomised groups (p=0·09; appendix p 16).

Resistance mutations were assayed in 58 (84%) of 69 children with viral load greater than 500 copies per mL at 96 weeks (19 allocated stavudine, 22 allocated zidovudine, and 17 allocated abacavir; remaining samples failed). Seven children (five allocated stavudine, one allocated zidovudine, and one allocated abacavir) had no NNRTI or NRTI mutations. As expected, M184V and NNRTI mutations were common in all groups, thymidine-analogue mutations (TAMs) were common in stavudine and zidovudine groups (although TAM-1 41L/210W/215Y were only seen in the zidovudine group), and 74V/115F mutations were common in the abacavir group (appendix p 18). However, only one K65R mutation was identified in the abacavir group. In the abacavir group, sensitivity to second-line NRTI options was 100% for zidovudine and 94% for tenofovir. In the zidovudine and stavudine groups, sensitivity to tenofovir remained high (86% and 100%, respectively; p=0·22 across randomised NRTIs; appendix p 21), but, as expected, was lower for their alternative second-line NRTI abacavir (64% and 89%, respectively; p=0·008 comparing susceptibility to the non-tenofovir second-line NRTI option across randomised groups).

## Discussion

In the first African paediatric trial comparing three NRTIs coformulated in NNRTI/NRTI generic fixed-dose-combination paediatric tablets, dosed using WHO drug ratios and weight bands,^2^, ^5^ we identified no major differences in any adverse event or toxicity endpoint during nearly 2·5 years follow-up in ART-naive and ART-experienced children. First-line drug substitutions occurred in only 6% of children, with nearly one-third due to starting anti-tuberculosis treatment. ART-naive children had good clinical, immunological, and virological responses, regardless of backbone NRTI; CD4 cell count and virological responses were maintained among almost all ART-experienced children. As expected, most deaths occurred early in children starting ART and only 1% switched to second-line therapy.

Paediatricians have long debated the relative advantages and disadvantages of different so-called backbone NRTIs combined with lamivudine, particularly because harmonising with adult tenofovir-based once-daily ART is not possible because of concerns about bone toxicity in growing children and absence of paediatric fixed-dose combinations or doses in those younger than 2 years. In the past decade, WHO guidelines have promoted paediatric fixed-dose combinations, first used in the CHAPAS-1 trial^18^ and licensed in 2007. However, preferred NRTI recommendations have changed from stavudine (2006) to zidovudine (2010^4^) to abacavir (2013^5^), based on minimal paediatric data and no randomised trials.

91% of children needing ART live in Africa, where genetic and environmental factors determine the relative effect of different ART toxicity profiles. We found no major differences across randomised NRTIs in grade 2–4 clinical or grade 3/4 laboratory adverse events, in either ART-naive or ART-experienced children. The only grade 3/4 event with marginally increased frequency was neutropenia in children allocated zidovudine; its significance is uncertain because African children have low neutrophil counts,^19^ and it rarely led to zidovudine substitution. As previously described,^20^ haemoglobin increased regardless of backbone NRTI, and severe anaemia occurred no more frequently in children who received zidovudine versus those who received stavudine or abacavir, suggesting HIV-related rather than drug-related cause. However, although infrequent, drug substitution was more common in the zidovudine group than both other groups, as was also reported in the ARROW trial,^20^ mainly for anaemia. These combined trial results reassure clinicians that zidovudine substitution is rarely needed for anaemia among children on ART. However, an important caveat is that severe anaemia and neutropenia were an exclusion criteria in both trials; if anaemia is HIV related, initiating zidovudine might also lead to good haemoglobin responses in anaemic children, as observed here, but we did not assess this.

Clinical lipodystrophy was not recorded up to 3 years follow-up of children aged younger than 5 years at ART initiation. Absence of blinding cannot rule out ascertainment bias, but lack of significant differences in body circumferences or skinfold thicknesses between NRTIs supports anecdotally reported rarity of lipodystrophy among young children, and suggests that longer-term consequences of stavudine exposure in young children are likely to be limited. We also found no evidence of a difference between NRTIs in changes in lipids on ART. Nevertheless, lipodystrophy undoubtedly occurs in older children and adolescents; the only lipodystrophy noted during the trial was facial in two older ART-experienced children already taking stavudine for more than 2·5 years. For this reason, and despite little evidence of harm in young children, the WHO 2013 recommendation that stavudine should be used only where other drugs are unavailable seems reasonable because it harmonises with adult and adolescent recommendations where evidence is strong. However, our results suggest that stavudine could be safely used for at least 2 years in young children (eg, with severe anaemia at ART initiation), if alternatives are not available, supporting WHO^5^ and the European Medicines Agency who recommended that stavudine for children should not be discontinued completely.

Despite no HLA-B5701 testing, no hypersensitivity reactions to abacavir were observed, in agreement with previous data reporting its rarity in African adults^21^ and children.^10^ The only three hypersensitivity reactions leading to a change in ART were substitutions from nevirapine to lopinavir plus ritonavir, albeit at a lower rate than in adults,^22^ consistent with previous paediatric reports.^18^ Reassuringly, and providing the first randomised data in children, a CHAPAS-3 substudy showed no difference in cardiovascular measurements or biomarkers between randomised NRTI groups.^23^, ^24^ One limitation is that our trial recruited more ART-naive and fewer ART-experienced children than was planned, reducing the power to detect differences between these subgroups, although no major interactions were identified.

When this trial was designed, the major questions related to toxicity profiles of the three NRTIs, with concerns over the potency of abacavir^12^, ^13^ only arising later. However, 478 children still provided good power to detect 10–15% differences in viral load suppression. CD4 recovery and retrospectively assayed viral load suppression to less than 100 copies per mL, less than 400 copies per mL, or less than 1000 copies per mL (data not shown) did not differ by randomised NRTI (appendix p 16). Overall suppression was better in ART-experienced than in ART-naive children, as expected, because ART-experienced children were suppressed at enrolment. Similarly to ARROW, there were no interactions suggesting differences in viral load suppression by NRTIs by age (<3 years *vs* >3 years),^20^ and, also in agreement with other reports, there was no evidence that viral load suppression depended on intrauterine nevirapine exposure beyond infancy^25^ or NNRTI in children older than 3 years.^26^ Although many seminal trials have been done in HIV-infected children by the IMPAACT/PACTG group, their randomised comparisons of combination therapy have focused on the third (non-NRTI) drug (eg, PACTG-1060 nevirapine *vs* lopinavir plus ritonavir), older drugs (eg, PACTG-327 didanosine), or on receiving an additional NRTI (eg, PACTG-300). No IMPAACT/PACTG trial has directly compared abacavir, zidovudine, or stavudine head-to-head within combination therapy. Our results differ from the only previous randomised, smaller trial of zidovudine versus abacavir (PENTA-5), which showed virological superiority of abacavir versus zidovudine over 5 years in children in well-resourced settings.^27^, ^28^ However, children received two NRTIs alone or with nelfinavir; with a potent third drug, as in CHAPAS-3, any superiority of abacavir over zidovudine could well be masked. Our results provide reassurance following recent observational analyses reporting poorer virological responses to abacavir versus stavudine in South African children.^12^, ^13^ Possible explanations for the difference include unmeasured confounding or drug–drug interactions between abacavir and lopinavir plus ritonavir (the standard third drug in South Africa).^29^ Of interest, we did not find that abacavir did worse in children with higher viral loads in CHAPAS-3, but only 24 ART-naive children were younger than 1 year, by contrast with the South African studies where many were younger than 1 year with high viral loads. The contribution of the fixed-dose combination rather than separate pills to virological success is difficult to estimate, but cannot affect our within-trial comparisons as all were using fixed-dose combinations.

Finally, these first randomised resistance data in African children on different NRTI plus NNRTI first-line ART reassuringly show that most children remained susceptible to second-line NRTIs over the medium term, regardless of initial NRTI. In particular, while those taking first-line zidovudine had significantly reduced susceptibility to abacavir second-line, those taking first-line abacavir retained high susceptibility to zidovudine; both retained high susceptibility to tenofovir, increasingly used in children older than 10 years who weigh more than 35 kg.

At trial closure (before trial results were known), all carers and children were offered continuing follow-up in the research trial centres (but without the transport refund provided by the trial) or moving to an ART programme site closer to where they lived. Children moving to ART programme sites were moved onto the ART regimen provided by the site (predominantly zidovudine at trial closure, abacavir for some Ugandan sites) to ensure that the ART programme site could continue to provide uninterrupted ART, in terms of drug provision and forecasting. Children staying at the research sites could continue their randomised regimen, because there was no reason to change drugs in children doing well and stable on a WHO recommended regimen, and being carefully followed for toxicity. However, although stavudine remains an option for children not able to take other NRTIs in 2010^4^ and 2013^5^ WHO guidelines, at trial closure Uganda national guidelines no longer recommended stavudine for children (previously, stavudine was an option for first-line ART in children <5 years). In Zambia, guidelines were based on duration on stavudine, with age being also used more recently. The recommended substitutions were therefore on a case-by-case basis. As a result of all these factors, as well as reduced demand for stavudine-based products by programmes (and hence scarcity from manufacturers), almost all children moved off stavudine at trial closure.

In conclusion, CHAPAS-3 shows primarily that children respond well to all NRTI/NNRTI recommended fixed-dose combinations in 2013 WHO guidelines with minimal drug toxicity. Most primary endpoints were morbid events, showing the very small contribution of antiretroviral toxicity to managing the HIV-infected child. The population was generally young, with early disease, and hence highly generalisable to increasing numbers entering ART programmes under universal treatment for those younger than 5 years. The fixed-dose combinations have different advantages and disadvantages in terms of number and frequency of tablets, cost, and availability as dual or triple drug fixed-dose combinations. Abacavir has very low toxicity in African children, a superior resistance profile for second-line NRTI sequencing, and is the only once-daily licensed NRTI fixed-dose combination (with lamivudine) for children, supporting its preferred use in first-line ART.^5^ Its only disadvantage is that it has a higher cost than zidovudine and stavudine (US$0·09 per baby tablet *vs* $0·05 for zidovudine and $0·03 for stavudine).^14^ A WHO survey in 2014 showed that paediatric use of abacavir was increasing (34%), whereas stavudine was decreasing (12%); zidovudine was 51% and also decreasing, thus data strongly arguing for further abacavir price reductions. Potential future triple abacavir-based combinations with efavirenz or dolutegravir could further simplify and improve durability of once-daily first-line ART for children who will need ART for much longer than adults.

## Figures and Tables

**Table 1:** Baseline characteristics

		**Naive**				**Experienced**			
		Stavudine (n=123)	Zidovudine (n=112)	Abacavir (n=130)	All (n=365)	Stavudine (n=33)	Zidovudine (n=46)	Abacavir (n=34)	All (n=113)
Centre UTH, Lusaka, Zambia		30 (24%)	25 (22%)	34 (26%)	89 (24%)	15 (45%)	22 (48%)	15 (44%)	52 (46%)
Baylor, Kampala, Uganda		42 (34%)	36 (32%)	41 (32%)	119 (33%)	7 (21%)	8 (17%)	7 (21%)	22 (19%)
JCRC, Kampala, Uganda		34 (28%)	36 (32%)	32 (25%)	102 (28%)	11 (33%)	16 (35%)	12 (35%)	39 (35%)
JCRC, Gulu, Uganda		17 (14%)	15 (13%)	23 (18%)	55 (15%)	0	0	0	0
Age (years)		2·6 (1·6–4·1)	2·6 (1·7–3·9)	2·7 (1·7–4·0)	2·6 (1·6–4·0)	6·5 (5·9–7·3)	6·0 (5·5–7·2)	5·9 (5·4–7·2)	6·2 (5·5–7·2)
Sex									
	Male	66 (54%)	54 (48%)	67 (52%)	178 (49%)	23 (70%)	22 (48%)	14 (41%)	59 (52%)
	Female	57 (46%)	58 (52%)	63 (48%)	187 (51%)	10 (30%)	24 (52%)	20 (59%)	54 (48%)
*Z* score									
	Weight-for-age	−2·3 (1·8)	−2·2 (1·6)	−1·9 (1·6)	−2·1 (1·6)	−1·1 (0·7)	−1·0 (1·2)	−1·4 (1·0)	−1·1 (1·0)
	Height-for-age	−2·5 (1·7)	−2·6 (1·7)	−2·3 (1·7)	−2·5 (1·7)	−1·6 (0·8)	−1·4 (1·2)	−1·8 (0·9)	−1·6 (1·0)
	Body-mass index-for-age	−0·7 (1·6)	−0·4 (1·4)	−0·5 (1·5)	−0·5 (1·5)	−0·1 (0·8)	−0·1 (1·0)	−0·3 (0·9)	−0·2 (0·9)
WHO stage*									
	1	17 (14%)	10 (9%)	14 (11%)	41 (11%)	8 (24%)	10 (22%)	7 (21%)	25 (22%)
	2	45 (37%)	46 (41%)	48 (37%)	139 (38%)	8 (24%)	9 (20%)	7 (21%)	24 (21%)
	3	50 (41%)	41 (37%)	56 (43%)	147 (40%)	8 (24%)	24 (52%)	11 (32%)	43 (38%)
	4	11 (9%)	15 (13%)	12 (9%)	38 (10%)	9 (27%)	3 (7%)	9 (26%)	21 (19%)
Viral load (copies per mL)†									
	Log_10_	5·6 (0·7)	5·4 (0·8)	5·3 (0·8)	5·4 (0·8)	<50	<50	<50	<50
	Absolute	328 320 (191 770–926 170)	252 390 (107 830–808 330)	217 540 (78 760–609 520)	270 670 (116 330–738 360)	<50	<50	<50	<50
	>100 000 copies per mL	100 (84%)	85 (79%)	95 (74%)	280 (79%)	0 (0%)	0 (0%)	0 (0%)	0 (0%)
CD4 cell count									
	CD4%	19% (12–23)	21% (15–26)	19% (11–24)	20% (13–25)	35% (28–39)	35% (30–40)	35% (31–39)	35% (30–39)
	Absolute CD4	865 (581– 1236)	925 (675–1434)	813 (490–1353)	893 (597–1299)	1143 (987–1414)	1164 (916–1641)	1362 (1072–1656)	1191 (962–1587)
Stavudine, years		..	..	..	..	3·0 (2·3–3·6)	3·9 (3·0–4·5)	3·5 (2·5–4·2)	3·5 (2·6–4·2)
Any pMTCT received by mother or child		15 (12%)	20 (18%)	21 (16%)	56 (15%)	3 (9%)	3 (7%)	3 (9%)	9 (8%)
Nevirapine‡ only		8 (7%)	12 (11%)	18 (14%)	38 (10%)	3 (9%)	3 (7%)	3 (9%)	9 (8%)
Nevirapine‡ and NRTI§		4 (3%)	3 (3%)	2 (2%)	9 (2%)	0	0	0	0
NRTI only§		3 (2%)	5 (4%)	1 (1%)	9 (2%)	0	0	0	0
Received nevirapine with randomised NRTIs in ART		87 (71%)	75 (67%)	90 (69%)	252 (69%)	29 (88%)	42 (91%)	30 (88%)	101 (89%)

Data are n (%), median (IQR), or mean (SD). UTH=University Teaching Hospital. JCRC=Joint Clinical Research Centre. pMTCT=prevention of mother-to-child transmission. NRTI=nucleoside reverse-transcriptase inhibitors. ART=antiretroviral treatment.

* Derived from pre-trial WHO event history before and after ART initiation in ART experienced.

† ART naive: four missing in stavudine group, four in zidovudine group, and two abacavir group due to samples not being stored.

‡ Single-dose nevirapine to either mother or child or both, or less than 2 days nevirapine to the child (with or without single-dose nevirapine to the mother).

§ Zidovudine to the child or zidovudine (majority) or zidovudine plus lamivudine to the mother.

**Table 2:** Primary and secondary endpoints (time to event)

		**Stavudine (n=156); N (%)**	**Zidovudine (n=157)**		**Abacavir (n=164)**			**Abacavir *vs* zidovudine; HR (95% CI)**
			N (%)	HR *vs* stavudine (95% CI)	N (%)	HR *vs* stavudine (95% CI)	p value*	
Primary endpoint adverse event†		104 (67%)	103 (65%)	0·99 (0·75–1·29)	105 (64%)	0·88 (0·67–1·15)	0·63	0·89 (0·68–1·17)
Specific subsets of primary endpoint adverse events								
	Anaemia, grade 3/4	5 (3%)	9 (6%)	1·93 (0·64–5·76)	6 (4%)	1·15 (0·35–3·78)	0·42	0·60 (0·21–1·69)
	Anaemia, grade 4	5 (3%)	7 (4%)	1·45 (0·46–4·57)	3 (2%)	0·57 (0·14–2·38)	0·38	0·39 (0·10–1·52)
	Neutropenia, grade 3/4	4 (3%)	12 (8%)	3·21 (1·03–9·98)	5 (3%)	1·21 (0·32–4·49)	0·04	0·38 (0·13–1·07)
	Neutropenia, grade 4	3 (2%)	10 (6%)	3·55 (0·97–12·9)	4 (2%)	1·29 (0·29–5·75)	0·06	0·36 (0·11–1·16)
	Hypersensitivity reaction‡	5 (3%)	1 (0·6%)	0·22 (0·03–1·86)	2 (1%)	0·38 (0·07–1·95)	0·21	1·75 (0·16–19·3)
	Lipodystrophy/lipoatrophy	2 (1%)	0	..	0	..	0·08	..
	Mitochondrial disease§	1 (0·6%)	0	..	1 (0·6%)	..	0·65	..
Grade 3/4 adverse events¶		46 (29%)	53 (34%)	1·24 (0·83–1·84)	51 (31%)	1·01 (0·68–1·50)	0·48	0·82 (0·56–1·20)
Grade 3/4 adverse events adjudicated as NRTI related‖		6 (4%)	12 (8%)	2·12 (0·79–5·66)	5 (3%)	0·80 (0·25–2·63)	0·10	0·38 (0·13–1·08)
Serious adverse events		46 (29%)	44 (28%)	0·98 (0·65–1·48)	42 (26%)	0·78 (0·51–1·19)	0·46	0·80 (0·52–1·22)
Serious adverse events adjudicated as NRTI related‖		8 (5%)	12 (12%)	1·50 (0·61–3·67)	6 (6%)	0·64 (0·22–1·85)	0·22	0·43 (0·16–1·14)
Toxicity causing ART modification**		4 (3%)	9 (6%)	2·24 (0·69–7·32)	1 (1%)	0·23 (0·03–2·09)	0·03	0·10 (0·01–0·83)
New WHO stage 3 or 4 event or death		9 (6%)	7 (4%)	0·84 (0·31–2·26)	13 (8%)	1·37 (0·58–2·30)	0·55	1·62 (0·65–4·07)
Death††		7 (4%)	3 (2%)	0·48 (0·12–1·85)	9 (5%)	1·23 (0·46–3·29)	0·35	2·56 (0·69–9·45)

All HRs were stratified for randomisation stratification factors. No evidence of interaction between naive versus experienced strata on any outcome in table 2 (p>0·1; 21 tests), except for serious adverse events (p=0·02; naive children with serious adverse events: 46 allocated stavudine, 40 allocated zidovudine, and 39 allocated abacavir; experienced children: none allocated stavudine, four allocated zidovudine, and three allocated abacavir; serious adverse events were most commonly lower respiratory tract infections or other specific infections). HR=hazard ratio. NRTI=nucleoside reverse-transcriptase inhibitors. ART=antiretroviral treatment.

* Stratified log-rank test.

† Clinical grade 2 or greater, laboratory grade 3 (confirmed), laboratory grade 4 (all).

‡ Includes grade 4 Stevens-Johnson syndrome from appendix p 7; adjudicated blind to actual NRTI received. Both children in the abacavir group continued abacavir without adverse effects. See appendix p 11 for details of adjudicated relation to antiretrovirals. One additional grade 1 hypersensitivity reaction (not a primary endpoint) also occurred in the abacavir group: again the child continued abacavir without adverse effects.

§ One cardiomyopathy (stavudine group) and one myopathy (abacavir group).

¶ Clinical grade 3 or greater, laboratory grade 3 (confirmed), laboratory grade 4 (all).

‖ See appendix p 4 for details of grade 3/4 adverse events and serious adverse events judged by the endpoint review committee as possibly having some relation to any of stavudine or zidovudine or abacavir (all events adjudicated for each drug blind to NRTI actually received, so toxicity from any of the three NRTIs could be attributed to each event). No grade 3/4 adverse events or serious adverse events were judged probably or definitely related.

** Not including changes for tuberculosis treatment (see main text); one toxicity substitution in child randomised to abacavir was actually from zidovudine to nevirapine for anaemia, following previous substitution of nevirapine to zidovudine (triple NRTI regimen) for tuberculosis treatment. Two stavudine and one zidovudine substitutions from nevirapine to lopinavir/ritonavir for rash/hypersensitivity reactions; all other substitutions were from stavudine or zidovudine.

†† See appendix p 15 for relation between NRTIs and deaths.

**Table 3:** Secondary endpoints (continuous)

	Stavudine change*	Zidovudine change*	**Difference**†**(95% CI)**	Abacavir change*	**Difference**†**(95% CI)**	**p value**‡	**Difference**†**(95% CI)**
**Growth**							
Weight-for-age	0·91	0·84	0·08 (−0·15 to 0·30)	0·75	−0·06 (−0·32 to 0·10)	0·21	−0·18 (−0·39 to 0·02)
Height-for-age	0·62	0·67	0·08 (−0·12 to 0·28)	0·61	0·02 (−0·21 to 0·24)	0·72	−0·06 (−0·27 to 0·15)
BMI-for-age	0·63	0·40	0·05 (−0·22 to 0·31)	0·42	−0·13 (−0·41 to 0·15)	0·40	−0·17 (−0·42 to 0·08)
**Body circumference and skinfolds**							
Waist:hip ratio	−0·03	−0·04	−0·01 (−0·02 to 0·00)	−0·05	−0·01 (−0·03 to 0·01)	0·33	0·00 (−0·01 to 0·02)
Waist:arm ratio	−0·03	−0·04	−0·04 (−0·10 to 0·00)	−0·05	0·03 (−0·03 to 0·09)	0·13	0·07 (0·00 to 0·13)
Torso:arm skinfold ratio	−0·02	−0·02	−0·00 (−0·04 to 0·03)	−0·03	0·01 (−0·03 to 0·05)	0·87	0·01 (−0·03 to 0·05)
Sum of four skinfolds (mm)	−1·88	−3·75	−1·13 (−2·86 to 0·60)	−2·70	−0·28 (−2·07 to 1·52)	0·42	0·86 (−1·00 to 2·72)

* Mean change from baseline at 96 weeks.

† Mean difference in change in first 96 weeks from generalised estimating equation model.

‡ Global test from generalised estimating equations with normally distributed errors and independent covariance. BMI=body-mass index.
