# Supplementary material for: Abacavir, zidovudine, or stavudine as paediatric tablets for African HIV-infected children (CHAPAS-3): an open-label, parallel-group, randomised controlled trial
Source: Lancet Infect Dis. 2016 Feb;16(2):169–79. doi: 10.1016/S1473-3099(15)00319-9 (PMC4726762; doi:10.1016/S1473-3099(15)00319-9)
Supplement: Supplementary appendix [file mmc1.pdf]

THE LANCET Infectious Diseases

Supplementary webappendix

This webappendix formed part of the original submission and has been peer reviewed. We post it as supplied by the authors.

Supplement to: Mulenga V, Musiime V, Kekitiinwa A, et al, on behalf of the CHAPAS-3 trial team. Abacavir, zidovudine, or stavudine as paediatric tablets for African HIV-infected children (CHAPAS-3): an open-label, parallel-group, randomised controlled trial. *Lancet Infect Dis* 2015; published online Oct 6. http://dx.doi.org/10.1016/S1473-3099(15)00319-9.

Supplementary material

Supplementary Methods....2

(a) Eligibility criteria....2

(b) Sample size calculation from the trial protocol....2

(c) Statistical methods....3

Supplementary Results....4

Supplementary Table 1 Primary endpoint adverse events: clinical grade 2/3/4, laboratory grade 4 or confirmed grade 3....7

Supplementary table 2 ERC attribution of relationship between hypersensitivity reactions and ART....11

Supplementary table 3 Body circumferences and skinfold thicknesses at enrolment and monthly change over 96 week follow-up (absolute values)....12

Supplementary table 4 Body circumference and skinfold thickness age-adjusted Z-scores at enrolment and monthly change over 96 week follow-up (absolute values)....13

Supplementary table 5 Mean change in lipids from baseline to 96 weeks....14

Supplementary table 6 Causes of death....15

Supplementary table 7 Viral load and CD4%....16

Supplementary table 8 NRTI resistance mutations at 96 weeks....18

Supplementary Figure 1 Monthly change in body circumferences and skinfold thicknesses from enrolment to week 96: differences between zidovudine vs stavudine and abacavir vs stavudine....19

Supplementary Figure 2 CD4 response....20

Supplementary Figure 3 Resistance at 96 weeks....21

Supplementary Methods

The CHAPAS-3 (Children with HIV in Africa - Pharmacokinetics and Adherence of Simple antiretroviral regimens) trial recruited children from Zambia (University Teaching Hospital, Lusaka) and Uganda (Baylor-Uganda Centre of Excellence, Kampala; Joint Clinical Research Centre, Kampala and Gulu (satellite site)).

(a) Eligibility criteria

Confirmed HIV-infected children aged 1 month to 13 years weighing 3-30kg were enrolled if they were: (1) previously untreated and met WHO 2010 criteria for ART (ART-naïve), or (2) on stavudine-containing ART for ≥2 years with screening viral load (VL) <50 copies/ml and stable CD4/CD4% (ART-experienced). ART-experienced children had to have no signs of lipodystrophy, and in Uganda ART-naïve children had to be <5 years old (following national guidelines for receiving stavudine). Children with acute infections, on medications contraindicated with ART (including tuberculosis treatment in those <3 years who would need to receive triple NRTI rather than nevirapine), unlikely to adhere or attend, with laboratory abnormalities contraindicating ART or perinatally exposed to ART (including single-dose nevirapine) and aged <1 year were ineligible.

(b) Sample size calculation from the trial protocol

Assuming that children were enrolled over 18 months and followed for a minimum of 2 years, cumulative lost-to-followup/ death was 20% at the end of the 3.5 year period, and incidence of the primary toxicity endpoint on d4T containing regimens was 20% per year (approximately twice the rate of adverse events leading directly to ART substitutions in adults[1]), then 470 children would provide at least 85% power to detect reductions in toxicity incidence to 10% per year (HR=0.50) across the three arms, and 80% power to detect reductions to 10.5% per year (HR=0.525). If the toxicity rates were in fact half this, ie only 10% per year, 470 children would provide at least 85% power to detect larger reductions in toxicity incidence to 3.7% per year (HR=0.37) across the three arms. We consider that if the true rate of the primary toxicity endpoint is only 10% per year in children, then such larger improvement would be needed to justify changing policy to recommending different first-line drugs.

Randomising a total of 470 children would provide at least 80% power to detect differences of 0.55 units between randomised arms within each strata in the change in age-adjusted skinfold thicknesses (assuming standard deviation for the change in z-score from baseline is 1.6,[2] and 25% missing measurements at 96 weeks from death/lost to follow-up or missing value). The entire group of 470 patients would provide 85% power to detect smaller differences of 0.4 units. Of note, the normal range (5th to 95th percentile) corresponds to a z-score of -2 to +2: so, for example a difference of 0.55 units is the difference between the 10th and 23rd percentile, or between the 25th and 40th percentile, differences which we consider to be clinically relevant.

(c) Statistical methods

Time-to-event analyses measured time from randomization, censored at the last clinical follow-up if the outcome had not occurred.

Weight, height, BMI, skinfold thicknesses, body circumferences and CD4 were compared between randomized groups over time using generalized estimating equations (GEE) (independent correlation structure) with randomized group, stratification factors and scheduled visit week as categorical independent variables, adjusting for baseline value. An interaction term was included between ART-naïve vs experienced and randomized group as this was the primary exposure over which effects of randomised group were considered might differ. The closest measurement to each scheduled visit date within equally spaced windows was used as the measurement at each scheduled visit. Continuous measurements were modeled using change from baseline as the outcome in a normal GEE model. Adherence measures were modeled as dichotomous outcomes in a binomial GEE model. Baseline values were those nearest to but before and within 42 days of randomization.

For all analyses global significance tests pool comparisons between randomised groups across strata, and heterogeneity tests assess whether differences between NRTIs vary between strata.

Weight/height-for-age Z-scores were calculated using UK 1990 reference data[3] which covers the full age range of CHAPAS-3 children. Body circumference and skinfold measurement Z-scores were calculated

using Dutch reference data[4, 5] as there are no African reference data; raw measurements were used in secondary analyses. Four composite measures were also considered, namely waist:hip, waist:MUAC, torso:arm ratios and the sum of subscapular, suprailiac, biceps and triceps skinfold thicknesses.[6]

Drug susceptibility was estimated from genotype according to the Stanford algorithm v7.0.

Supplementary Results

For the primary endpoint (figure 2a) there were 835 events in 269(74%) ART-naïve children versus 82 events in 43(38%) ART-experienced children. For serious adverse events, most events (163/199, 82%) caused or prolonged hospitalisation, and the most common diagnoses were pneumonia (67), malaria (34) and septicaemia/bacteraemia (24).

The Endpoint Review Committee (ERC) adjudicated the relationship of each primary endpoint to each of stavudine, zidovudine and abacavir blind to the NRTI actually received, so toxicity from any of the three NRTIs could be attributed to each event. Of note, whilst this objectively assigned specific toxicities to events (eg anaemia of a similar severity would be classified as possibly zidovudine-related regardless of NRTI received), it also meant that the child might not be receiving the drug adjudicated as possibly related by the ERC.

No grade 3/4 AEs were judged by the ERC as probably/definitely related to any of stavudine, zidovudine or abacavir. Grade 3/4 AEs judged by the ERC as possibly related to at least one of stavudine, zidovudine or abacavir were as follows: stavudine group – death, kwashiorkor, fat wasting, clinical anaemia, clinical neutropenia, other gram-negative sepsis; zidovudine group – death, Stevens-Johnson syndrome, *P. falciparum* malaria, pancytopenia, clinical anaemia (3), clinical neutropenia (5); abacavir group – abdominal tuberculosis, pneumococcal septicaemia, clinical anaemia, clinical neutropenia (2).

No SAEs were judged by the ERC as probably/definitely related to any of stavudine, zidovudine or abacavir. SAEs judged by the Endpoint Review Committee as possibly related to at least one of stavudine, zidovudine or abacavir were as follows: stavudine group - 4 deaths (cardiomyopathy, kwashiorkor,

pneumonia, cause unknown), 1 hospitalisation (clinical anaemia), 3 other events (upper respiratory tract infection (URTI), 2 hypersensitivity reactions); zidovudine group – 1 death (septicaemia/bacteraemia), 2 life-threatening (2 clinical anaemia) 7 hospitalisations (Stevens Johnson syndrome, 2 URTI, p falc malaria, pneumonia, septicaemia/bacteraemia, clinical anaemia), other events (clinical anaemia, clinical neutropenia); abacavir group – 1 death (pneumonia), 1 life threatening (septicaemia/bacteraemia), 3 hospitalisations (*P. falciparum* malaria, pneumonia, septicaemia/bacteraemia), 1 other event (hypersensitivity reaction).

The three children who stopped nevirapine following hypersensitivity reactions were all ART naïve at randomisation and aged between 18 months and 3 years. The reactions occurred between two and four weeks after ART initiation; in each case nevirapine was substituted with lopinavir/ritonavir and the child then continued in the trial until study closure.

There were few differences between randomised groups in changes in body circumferences and skinfold thicknesses, and those that occurred were not consistent in their direction between randomised groups (supplementary tables 2 and 3, supplementary figure 1). Increases in calf circumference were smaller in children on abacavir (p=0.01); reduction in supra-iliac skinfold was greater in naïve children on zidovudine and in experienced children on abacavir (p=0.01).

Weight gain was rapid among ART-naïve children (mean weight-for-age Z-score greater than -1 in all randomised groups at 48 weeks), as was CD4% recovery, with mean CD4% above 30% in all groups at 48 weeks (supplementary table 4).

SUPPLEMENTARY REFERENCES

1. Boulle A, Orrel C, Kaplan R, et al. Substitutions due to antiretroviral toxicity or contraindication in the first 3 years of antiretroviral therapy in a large South African cohort. *Antiviral therapy* 2007; 12(5): 753-60.

2. Dzwonek AB, Lawson MS, Cole TJ, Novelli V. Body fat changes and lipodystrophy in HIV-infected children: impact of highly active antiretroviral therapy. *Journal of acquired immune deficiency syndromes* 2006; 43(1): 121-3.

3. Wade AM, Ades AE. Age-related reference ranges: significance tests for models and confidence intervals for centiles. *Statistics in medicine* 1994; 13(22): 2359-67.

4. Fredriks AM, van Buuren S, Fekkes M, Verloove-Vanhorick SP, Wit JM. Are age references for waist circumference, hip circumference and waist-hip ratio in Dutch children useful in clinical practice? *European journal of pediatrics* 2005; 164(4): 216-22.

5. Gerver WJM, de Bruin R. Paediatric Morphometrics: A Reference Manual Maastricht: Universitaire Pers Maastricht; 2001.

6. Innes S, Levin L, Cotton M. Lipodystrophy Syndrome in Hiv-Infected Children on Haart. *Southern African journal of HIV medicine* 2009; 10(4): 76-80.

Supplementary Table 1 Primary endpoint adverse events: clinical grade 2/3/4, laboratory grade 4 or confirmed grade 3

	Stavudine	Zidovudine	Abacavir	All
Total number of grade 2/3/4 AEs	285	298	334	917
Clinical	275	282	320	877
Grade 2	188	202	244	634
Grade 3	73	65	54	192
Grade 4	14	15	22	51
Laboratory	10	16	14	40
Grade 3 confirmed	0	3	5	8
Grade 4	10	13	9	32
**Total Grade 4 events**	**24**	**28**	**31**	**83**
Laboratory – haematology				
Neutrophils	3	7	1	11
Haemoglobin	4	3	3	10
Laboratory – biochemistry				
Raised liver enzymes			2	2
ALT	1	1	1	3
Bilirubin	1	1	1	3
Creatinine	1	1	1	3
Haematological				
Pancytopenia - bone marrow depression	0	1	0	1
Anaemia with clinical symptoms	2	5	2	9
Neutropenia with clinical symptoms	0	3	3	6
Tumours				
Kaposi's sarcoma lymph nodes	0	0	1	1
CNS				
Epilepsy - fits - convulsions	0	0	1[a]	1
Cardiovascular disease				
Cardiomyopathy	1[a]	0	0	1
Diarrhoeal disease				
Acute diarrhoea, idiopathic AIDS enteropathy	2	0	0	2
Gastrointestinal				
Gastroenteritis	0	1	0	1
Hepatic				
Acute hepatitis	0	0	2	2
Lower respiratory tract				
Aspiration pneumonia	0	0	1[a]	1
Pneumonia - Pneumococcal	0	0	1	1
Pneumonia - other bacterial	1[a]	0	0	1
Pneumonia no organism identified	1	1[a]	2[c]	4
Upper respiratory disease				
Other acute upper respiratory symptoms	0	0	1	1
Systemic				
Malnutrition	0	0	1	1
Stevens-Johnson Syndrome*	0	1	0	1
Kwashiorkor	1[a]	0	1[a]	2
Specific Infections				
Tuberculosis - abdominal	0	0	1	1
P falciparum malaria	0	1	0	1
Measles	0	0	2[b]	2
Presumed septicaemia/bacteremia, uninvestigated	0	2[c]	1[a]	3
Pneumococcal septicaemia	0	0	1[a]	1
Presumed septicaemia/bacteremia, no organism	1[a]	0	0	1
Other gram negative sepsis	2	0	0	2
Death - cause unknown	3	0	1	4

	Stavudine	Zidovudine	Abacavir	All
**Total Grade 3 events**	**73**	**68**	**59**	**200**
Laboratory – haematology				
Haemoglobin	0	1	3	4
Neutrophils	0	2	1	3
Laboratory – biochemistry				
ALT	0	0	1	1
Haematological				
Neutropenia with clinical symptoms	1	2	0	3
Persistent generalised lymphadenopathy	0	1	0	1
Anaemia with clinical symptoms	0	1	0	1
Biochemical				
Hyperglycaemia	1	0	0	1
CNS				
Epilepsy, fits, convulsions	0	2	1	3
Pyogenic meningitis, no organism	0	1	0	1
Stroke, cerebrovascular accident	1	0	0	1
Diarrhoeal disease				
Chronic diarrhoea with cryptosporidia	0	0	1	1
Chronic diarrhoea not investigated	1	0	0	1
Acute diarrhoea, idiopathic AIDS enteropathy	6	1	1	8
Acute diarrhoea with other pathogen	0	1	0	1
Acute diarrhoea not investigated	0	0	1	1
Gastrointestinal				
Vomiting	1	0	0	1
Renal				
Lower urinary tract infection, cystitis	1	0	0	1
Lower respiratory tract				
Tuberculosis, pulmonary, smear +	0	1	1	2
Tuberculosis, pulmonary, smear -/not done	2	1	0	3
Pneumonia, Pneumococcal	1	0	0	1
Pneumonia, other bacterial	2	0	1	3
Pneumonia other organism	0	0	1	1
Pneumonia no organism identified	19	14	19	52
Bronchospasm/Asthma	0	2	0	2
Bronchiolitis	1	0	0	1
Chest infection	5	0	3	8
Upper respiratory disease				
URTI, not sinusitis or otitis media, acute	1	0	0	1
Chronic otitis media	0	0	1	1
Acute pharyngitis	0	2	0	2
Systemic				
Hypersensitivity reaction*	1	0	0	1
Kwashiorkor	1	1	2	4
Malnutrition	0	0	2	2
Fat wasting or loss of fat	1	0	0	1
Specific Infections				
P falciparum malaria	5	18	11	34
Other malaria	2	0	0	2
Tuberculosis, disseminated/miliary	1	0	0	1
Tuberculosis, lymph nodes	0	1	0	1
Measles	1	3	1	5
Salmonella bacteraemia – non-typhi	0	1	0	1
Pneumococcal septicaemia	1	0	0	1
Presumed septic/bacteremia, no organism	4	4	2	10
Presumed septic/bacteremia, uninvestigated	7	2	4	13
Other gram negative sepsis	0	1	0	1
Skin				
Rash, erythematous	0	0	1	1
Tinea, athletes foot, fungal infection of foot/skin	1	0	0	1
Cellulitis	1	1	0	2
Undiagnosed fevers				
Acute febrile episode, undiagnosed	1	1	0	2
Oral				

	Stavudine	Zidovudine	Abacavir	All
Acute parotitis	0	1	0	1
Herpes Simplex ulceration, oral	1	2	0	3
Musculoskeletal				
Septic arthritis	1	0	0	1
Arthralgia, arthritis, joint pain, arthropathies	1	0	0	1
Other				
Non-fatal trauma	0	0	1	1
**Total Grade 2 events**	**188**	**202**	**244**	**634**
Haematological				
Anaemia with clinical symptoms	0	0	1	1
Lymphadenopathy	2	2	1	5
Neutropenia with clinical symptoms	1	1	0	2
CNS				
Cranial nerve lesion	0	1	0	1
Epilepsy, fits, convulsions	0	0	1	1
Diarrhoeal disease				
Chronic diarrhoea with cryptosporidia	0	0	1	1
Chronic diarrhoea with no pathogen	1	0	0	1
Chronic diarrhoea with giardia	2	0	0	2
Acute diarrhoea with other pathogen	1	1	0	2
Acute diarrhoea not investigated	4	7	9	20
Acute diarrhoea - idiopathic AIDS enteropathy	13	11	6	30
Gastrointestinal				
Indigestion, oesophageal reflux, gastritis	0	1	0	1
Vomiting	0	0	1	1
Hepatic				
Hepatitis A	0	1	0	1
Renal				
Lower urinary tract infection - cystitis	3	1	1	5
Lower respiratory tract				
Bronchospasm/Asthma	0	0	1	1
Pneumonia, other bacterial	1	0	0	1
Pneumonia, Pneumococcal	1	0	2	3
Pneumonia no organism identified	19	12	9	40
Chest infection	38	36	45	119
Upper respiratory disease				
URTI, not sinusitis or otitis media, chronic	1	0	0	1
URTI, not sinusitis or otitis media, acute	34	43	83	160
Chronic otitis media	7	7	12	26
Acute otitis media	11	20	16	47
Otitis externa	0	4	2	6
Acute pharyngitis	7	8	6	21
Systemic				
Hypersensitivity reaction*	4	0	2	6
Malnutrition	0	2	1	3
Fat wasting or loss of fat	1	0	0	1
Specific Infections				
P falciparum malaria	3	5	6	14
Measles	5	3	0	8
Mumps	0	0	1	1
Tonsilitis	0	1	0	1
Presumed septic/bacteremia, no organism	1	0	0	1
Presumed septic/bacteremia, not investigated	6	4	9	19
Other gram positive sepsis	1	0	0	1
Skin				
Herpes Zoster (Varicella Zoster), cutaneous	1	1	2	4
Chicken Pox	0	1	1	2
Rash, maculopapular	0	0	1	1
Itching, pruritis, excoriation, scratching	0	0	1	1
Skin abscess	1	0	1	2
Tinea, athletes foot, fungal infection of foot/skin	2	3	2	7
Cellulitis	2	1	4	7
Folliculitis, furuncles, carbuncles	3	6	4	13

	Stavudine	Zidovudine	Abacavir	All
Impetigo	3	0	1	4
Undiagnosed fevers				
Febrile convulsions	0	0	1	1
Acute febrile episode, undiagnosed	3	5	2	10
Genitourinary				
Vulvovaginal candidiasis	3	0	1	4
Oral				
Herpes Simplex ulceration, oral	0	6	1	7
Acute parotitis	1	3	1	5
Oral candida	1	2	0	3
Dental abscess	0	0	1	1
Eye				
Conjunctivitis, purulent/bacterial	0	1	3	4
Other				
Overdose (not suicide attempt)	0	1	0	1
Burns	1	1	1	3

a. fatal event, b. includes 1 fatal event, c. 2 fatal events

* see supplementary table 2 for ERC adjudication of relationship between hypersensitivity reactions and ART

Supplementary table 2 ERC attribution of relationship between hypersensitivity reactions and ART

	Event	Grade	NRTI possibly related*	Other antiretroviral possibly related	Other antiretroviral probably or definitely related
Stavudine group					
	Hypersensitivity reaction	3		**nevirapine**	
	Hypersensitivity reaction	2	abacavir	**nevirapine**, efavirenz	
	Hypersensitivity reaction	2	abacavir	nevirapine, **efavirenz**	
	Hypersensitivity reaction	2			
	Hypersensitivity reaction	2	abacavir	**nevirapine**, efavirenz	
Zidovudine group					
	Stevens Johnson	4	abacavir		**nevirapine**
Abacavir group					
	Hypersensitivity reaction	2	**abacavir ****	**nevirapine**, efavirenz	
	Hypersensitivity reaction	2	**abacavir ****	**nevirapine**, efavirenz	

* no NRTI was adjudicated probably/definitely related to a hypersensitivity reaction

** did not stop abacavir, no adverse consequences

Note: The ERC adjudicated relationships to each antiretroviral drug without knowing which the child was randomised to or received. Antiretrovirals which the child had actually taken are underlined in bold text.

Supplementary table 3 Body circumferences and skinfold thicknesses at enrolment and monthly change over 96 week follow-up (absolute values)

	Baseline measurement						Monthly change		Difference in change		Difference in change		
	stavudine		zidovudine		abacavir		stavudine		zidovudine-stavudine		abacavir-stavudine		
ART naïve	mean	(sd)	mean	(sd)	mean	(sd)	mean	(95%CI)	mean	(95%CI)	mean	(95%CI)	p[†]
waist[a]	48.5	(5.8)	48.9	(5.6)	49.7	(5.3)	0.12	(0.09,0.15)	-0.011	(-0.054,0.033)	-0.016	(-0.058,0.026)	0.06
hip[a]	46.8	(6.2)	47.2	(6.1)	48.4	(6.9)	0.19	(0.16,0.23)	-0.004	(-0.051,0.044)	-0.005	(-0.057,0.047)	0.22
muac[a]	14.8	(1.8)	14.8	(1.8)	15.1	(1.7)	0.05	(0.03,0.06)	0.001	(-0.017,0.019)	-0.009	(-0.027,0.008)	0.10
thigh[a]	25.2	(3.8)	25.4	(4.0)	25.9	(3.8)	0.12	(0.10,0.14)	-0.008	(-0.043,0.028)	-0.010	(-0.046,0.026)	0.44
calf[a]	17.8	(2.7)	17.8	(2.7)	18.4	(2.7)	0.10	(0.08,0.11)	0.004	(-0.017,0.026)	-0.011	(-0.030,0.008)	0.01
bicep[b]	5.8	(1.7)	6.2	(1.4)	6.0	(1.5)	0.00	(-0.02,0.02)	-0.007	(-0.037,0.024)	0.011	(-0.021,0.043)	0.07
tricep[b]	7.9	(2.5)	8.6	(2.2)	8.1	(2.1)	0.00	(-0.02,0.02)	-0.016	(-0.052,0.019)	0.004	(-0.029,0.038)	0.26
thigh[b]	10.7	(3.6)	11.8	(3.0)	11.2	(3.3)	0.01	(-0.03,0.04)	-0.037	(-0.086,0.013)	0.005	(-0.051,0.061)	0.11
scapular[b]	5.9	(1.9)	6.2	(1.7)	6.1	(1.7)	-0.01	(-0.03,0.00)	-0.017	(-0.043,0.007)	-0.002	(-0.027,0.022)	0.30
supra-iliac[b]	4.7	(1.8)	5.5	(2.3)	5.1	(1.9)	-0.02	(-0.03,0.00)	-0.025	(-0.045,-0.004)	-0.001	(-0.022,0.020)	0.01
ART experienced													
waist[a]	54.6	(3.8)	54.5	(3.4)	54.7	(3.8)	0.11	(0.08,0.15)	-0.022	(-0.074,0.030)	-0.065	(-0.110,-0.020)	
hip[a]	57.4	(4.0)	57.1	(3.8)	55.8	(4.1)	0.15	(0.10,0.20)	0.061	(-0.004,0.125)	-0.006	(-0.062,0.051)	
muac[a]	16.9	(1.5)	16.6	(1.1)	16.9	(1.3)	0.03	(0.02,0.05)	0.011	(-0.014,0.035)	-0.020	(-0.046,0.005)	
thigh[a]	30.8	(2.9)	30.6	(2.7)	30.1	(2.8)	0.11	(0.08,0.14)	0.014	(-0.035,0.063)	-0.027	(-0.071,0.016)	
calf[a]	22.4	(1.9)	22.1	(1.6)	21.9	(1.7)	0.08	(0.06,0.10)	0.003	(-0.020,0.028)	-0.026	(-0.047,-0.005)	
bicep[b]	4.8	(1.3)	5.0	(1.4)	4.9	(1.5)	-0.07	(-0.08,-0.05)	-0.012	(-0.031,0.007)	-0.027	(-0.046,-0.008)	
tricep[b]	6.7	(1.9)	6.7	(1.6)	6.6	(1.7)	-0.09	(-0.11,-0.07)	-0.007	(-0.036,0.022)	-0.031	(-0.063,0.001)	
thigh[b]	8.4	(2.6)	8.7	(2.7)	8.3	(2.5)	-0.10	(-0.15,-0.05)	-0.018	(-0.068,0.032)	-0.055	(-0.108,-0.001)	
scapular[b]	5.7	(1.3)	5.6	(1.4)	5.9	(1.6)	-0.06	(-0.08,-0.05)	0.004	(-0.018,0.026)	-0.013	(-0.033,0.007)	
supra-iliac[b]	4.6	(1.3)	4.7	(1.6)	5.0	(1.7)	-0.05	(-0.07,-0.04)	0.007	(-0.012,0.027)	-0.016	(-0.033,0.001)	

a, body circumference in centimetres. b, skinfold thickness in millimetres.

† overall significance test of difference between randomised arms in the change over 2 years, adjusted for age, sex and ART naïve/experienced strata

Supplementary table 4 Body circumference and skinfold thickness age-adjusted Z-scores at enrolment and monthly change over 96 week follow-up (absolute values)

	Baseline measurement						Monthly change		Difference in change		Difference in change		
	stavudine		zidovudine		abacavir		stavudine		zidovudine-stavudine		abacavir-stavudine		
ART naïve	mean	(sd)	mean	(sd)	mean	(sd)	mean	(95%CI)	mean	(95%CI)	mean	(95%CI)	p[†]
waist[a]	0.2	(1.5)	0.4	(1.4)	0.4	(1.3)	-0.004	(-0.013,0.006)	-0.001	(-0.015,0.013)	-0.005	(-0.017,0.008)	0.36
hip[a]	-1.0	(1.2)	-0.8	(1.3)	-0.7	(1.3)	0.003	(-0.007,0.012)	0.001	(-0.012,0.015)	-0.001	(-0.015,0.012)	0.49
muac[a]	-1.6	(1.3)	-1.6	(1.3)	-1.5	(1.2)	0.007	(-0.003,0.017)	0.003	(-0.012,0.017)	-0.009	(-0.022,0.004)	0.10
thigh[a]	-1.8	(1.4)	-1.7	(1.3)	-1.7	(1.5)	-0.001	(-0.011,0.009)	-0.001	(-0.016,0.014)	-0.005	(-0.019,0.009)	0.29
calf[a]	-2.4	(1.5)	-2.4	(1.5)	-2.2	(1.4)	0.009	(-0.001,0.018)	0.006	(-0.010,0.022)	-0.006	(-0.018,0.007)	0.08
bicep[b]	-0.3	(1.0)	-0.1	(0.8)	-0.2	(0.8)	0.004	(-0.008,0.015)	-0.004	(-0.021,0.013)	0.006	(-0.012,0.024)	0.01
tricep[b]	-0.5	(1.1)	-0.3	(1.0)	-0.5	(0.9)	-0.005	(-0.016,0.006)	-0.002	(-0.019,0.016)	0.005	(-0.011,0.021)	0.19
scapular[b]	0.0	(1.3)	0.2	(1.3)	0.1	(1.2)	-0.010	(-0.025,0.004)	-0.006	(-0.027,0.015)	0.002	(-0.018,0.021)	0.20
supra-iliac[b]	-0.4	(1.0)	-0.1	(1.3)	-0.3	(1.1)	-0.022	(-0.031,-0.013)	-0.008	(-0.022,0.007)	0.005	(-0.007,0.018)	0.05
ART experienced													
waist[a]	0.1	(0.8)	0.1	(0.9)	0.1	(0.9)	-0.025	(-0.035,-0.015)	-0.003	(-0.017,0.011)	-0.012	(-0.024,0.001)	
hip[a]	-0.7	(0.6)	-0.7	(1.0)	-1.1	(0.9)	-0.031	(-0.044,-0.019)	0.013	(-0.004,0.029)	0.000	(-0.016,0.016)	
muac[a]	-1.1	(0.9)	-1.4	(1.0)	-1.2	(1.0)	-0.007	(-0.020,0.005)	0.006	(-0.011,0.024)	-0.015	(-0.035,0.005)	
thigh[a]	-2.0	(0.9)	-2.0	(1.1)	-2.3	(1.0)	-0.022	(-0.033,-0.011)	0.001	(-0.015,0.017)	-0.012	(-0.026,0.001)	
calf[a]	-1.8	(0.9)	-1.7	(1.2)	-1.9	(0.9)	-0.005	(-0.017,0.007)	0.002	(-0.013,0.017)	-0.012	(-0.026,0.001)	
bicep[b]	-0.3	(0.8)	-0.4	(0.9)	-0.5	(1.0)	-0.023	(-0.034,-0.012)	-0.015	(-0.030,0.000)	-0.027	(-0.041,-0.013)	
tricep[b]	-0.9	(0.8)	-1.1	(0.7)	-1.1	(0.8)	-0.033	(-0.043,-0.023)	-0.006	(-0.019,0.007)	-0.018	(-0.032,-0.003)	
scapular[b]	0.3	(0.9)	0.0	(1.2)	0.2	(1.3)	-0.039	(-0.051,-0.028)	-0.004	(-0.020,0.012)	-0.017	(-0.032,-0.002)	
supra-iliac[b]	-1.0	(0.8)	-1.0	(0.9)	-0.8	(1.1)	-0.044	(-0.052,-0.035)	0.000	(-0.009,0.010)	-0.011	(-0.020-,0.001)	

a, body circumference in centimetres. b, skinfold thickness in millimetres.

† overall significance test of difference between randomised arms, change over 2 years, adjusted for age, sex and ART naïve/experienced

Supplementary table 5 Mean change in lipids from baseline to 96 weeks

	Stavudine (n=156)	Zidovudine (n=157)		Abacavir (n=164)			Abacavir vs. zidovudine
	change†	change†	diff†† (95%CI)	change†	diff†† (95%CI)	p**	diff†† (95%CI)
Lipids (mmol/l)							
Total cholesterol	0.76	0.49	-0.23 (-0.59,0.14)	0.58	-0.19 (-0.55,0.18)	0.43	0.04 (-0.31,0.40)
LDL	0.36	0.25	-0.07 (-0.28,0.15)	0.35	0.00 (-0.21,0.21)	0.76	0.07 (-0.14,0.28)
HDL	0.50	0.48	0.01 (-0.14,0.17)	0.46	-0.04 (-0.19,0.12)	0.80	-0.05 (-0.20,0.10)
Triglycerides	-0.37	-0.21	0.13 (-0.14,0.40)	-0.28	0.09 (-0.18,0.36)	0.62	-0.04 (-0.30,0.22)

† mean change from baseline at 96 weeks

†† mean difference in change over first 96 weeks from generalised estimating equation model

Supplementary table 6 Causes of death

	At enrolment				ERC adjudication	
Weeks in trial	Age (years)	CD4%	CD4	Sex	Cause of death	NRTIs possibly related*
Stavudine group						
1.7	1.3	4%	195	M	Unknown (died at home - malnutrition, probable pneumonia)	
4.9	2.6	16%	1008	M	Unknown (died on way to hospital - sudden onset respiratory distress)	Zidovudine, **Stavudine**
5.0	4.3	5%	156	F	Pneumonia, malnutrition, hyperglycaemia, gram negative sepsis	Zidovudine
6.4	1.7	20%	1188	F	Kwashiorkor, neutropenia, gram negative sepsis	Zidovudine
10.7	12.0	2%	44	M	Cardiomyopathy	Zidovudine, **Stavudine**
23.1	0.7	16%	1337	M	Septicaemia/bacteraemia	
94.1	1.7	2%	46	F	Unknown (died at home – malnutrition, refused hospital admission)	
Zidovudine group						
4.6	3.9	12%	621	F	Malaria, anaemia, septicaemia/bacteraemia	**Zidovudine**
5.4	1.9	14%	838	M	Pneumonia	
14.7	1.9	13%	904	F	Marasmus, gastroenteritis, septicaemia/bacteraemia	
Abacavir group						
6.0	0.7	11%	597	M	Pneumonia, marasmus	
11.1	1.7	16%	774	F	Unknown (died at home - kwashiorkor, carer took from hospital to traditional healer)	
23.7	12.2	0%	4	M	Septicaemia/bacteraemia	
25.1	3.0	7%	82	F	Measles	
33.7	4.4	4%	63	M	Pneumonia, neutropenia, pneumococcal septicaemia	Zidovudine
38.1	1.3	14%	1732	M	Measles, pneumonia	
40.9	1.2	18%	1244	F	Kwashiorkor	
44.7	2.0	20%	1605	M	Epilepsy/fits, rash (died on way to hospital – probable measles)	
56.0	5.0	0%	16	M	Kaposi's sarcoma	

* no ART was adjudicated probably/definitely related to death

Note: all deaths occurred in children who were ART-naïve at enrolment. The ERC adjudicated relationships to each NRTI without knowing which the child was randomised to or received. NRTIs which the child had actually taken before dying are underlined in bold text. Relationship with zidovudine considered at least possible where septicaemia/bacteraemia was accompanied by neutropenia.

Supplementary table 7 Viral load and CD4%

ART naïve							
		n(%)				difference (95%CI)	
Tested	stavudine	zidovudine	abacavir	p	zidovudine-stavudine	abacavir-stavudine	abacavir-zidovudine
Week 0	119	108	128				
Week 48	115	101	117				
Week 96	106	102	111				
Week 144	24	29	30				
< 100 copies/ml							
Week 0	0 (0%)	1 (1%)	2 (2%)				
Week 48	77 (67%)	74 (73%)	83 (71%)	0.61	+6.3% (-5.9,18.5)	+4.0% (-7.9,15.9)	-2.3% (-14.3,9.6)
Week 96	77 (73%)	76 (75%)	85 (77%)	0.79	+1.9% (-10.1,13.8)	+3.9% (-7.6,15.5)	+2.1% (-9.5,13.6)
Week 144	20 (83%)	20 (69%)	26 (87%)	0.28	-14.4% (-36.9,8.1)	+3.3% (-15.9,22.6)	+17.7% (-3.1,38.5)
<400 copies/ml							
Week 0	0 (0%)	1 (1%)	3 (2%)				
Week 48	98 (85%)	81 (80%)	95 (81%)	0.58	-5.0% (-15.1,5.1)	-4.0% (-13.6,5.6)	+1.0% (-9.5,11.5)
Week 96	80 (75%)	78 (76%)	91 (82%)	0.46	+1.0% (-10.6,12.6)	+6.5% (-4.4,17.4)	+5.5% (-5.4,16.4)
Week 144	20 (83%)	20 (69%)	27 (90%)	0.13	-14.4% (-36.9,8.1)	+6.7% (-11.7,25.0)	+21.0% (1.1,41.0)
CD4%		mean (sd)				difference (95%CI) change from week 0	
Week 0	18.9 (10.4)	20.9 (8.5)	19.7 (11.7)				
Week 48	32.9 (12.1)	35.3 (10.4)	32.8 (12.9)	0.09*	+1.5 (0.1,3.0)	+0.1 (-1.3, 1.5)	-1.4 (-2.8,0.0)
Week 96	36.3 (10.8)	38.0 (9.6)	36.7 (12.6)	0.20*	+1.2 (-0.2,2.6)	+0.1 (-1.2,1.4)	-1.1 (-2.5,0.3)
Week 144	35.6 (6.6)	35.7 (7.5)	34.1 (9.1)	0.41*	+0.9 (-0.5,2.3)	+0.1 (-1.2,1.4)	-0.8 (-2.2,0.6)

ART experienced							
		n(%)				difference (95%CI)	
Tested	stavudine	zidovudine	abacavir	p	zidovudine-stavudine	abacavir-stavudine	abacavir-zidovudine
Week 48	32	45	34				
Week 96	32	45	32				
Week 144	15	21	14				
< 100 copies/ml							
Week 48	31 (97%)	40 (89%)	33 (97%)	0.33	-8.0% (-19.0,3.0)	+0.2% (-8.1,8.5)	+8.2% (-2.6,19.0)
Week 96	30 (94%)	44 (98%)	31 (97%)	0.82	+4.0% (-5.4,13.5)	+3.1% (-7.2,13.5)	-0.9% (-8.3,6.5)
Week 144	15 (100%)	18 (86%)	14 (100%)	0.11	-14.3% (-29.3,0.7)	0% (-23.0,22.0)	+14.3% (-0.7,29.3)
<400 copies/ml							
Week 48	31 (97%)	43 (96%)	33 (97%)	1.0	-1.3% (-9.8,7.2)	+0.2% (-8.1,8.5)	+1.5% (-6.8,9.8)
Week 96	31 (97%)	45 (100%)	31 (97%)	0.51	+3.1% (-2.9,9.2)	0% (-8.5,8.5)	-3.1% (-9.2,2.9)
Week 144	15 (100%)	19 (90%)	14 (100%)	0.50	-9.5% (-22.1,3.0)	0% (-23.0,22.0)	+9.5% (-3.0,22.1)
						difference (95%CI)	
CD4%		mean (sd)				change from week 0	
Week 0	33.9 (7.0)	34.6 (8.6)	34.0 (7.6)				
Week 48	33.0 (7.4)	34.8 (9.8)	34.2 (8.1)	0.39*	+1.7 (-1.0,4.4)	+1.8 (-1.0,4.5)	0.0 (-2.1,2.1)
Week 96	35.9 (8.8)	36.4 (8.4)	36.2 (6.2)	0.69*	+1.1 (-1.4,3.6)	+0.6 (-2.0,3.2)	-0.5 (-2.5,1.5)
Week 144	32.3 (5.7)	35.1 (11.1)	31.9 (13.0)	0.68*	+1.0 (-1.4,3.4)	+0.5 (-2.0,3.0)	-0.5 (-2.5,1.4)

* p-value for difference in change from week 0

Supplementary table 8 NRTI resistance mutations at 96 weeks

	Stavudine (N=19)	Zidovudine (N=22)	Abacavir (N=17)	Total (N=58)
NRTI mutations				
None	6	3	2	11
184V	11	10	5	26
65R 184I	0	0	1	1
70E 184V	0	0	1	1
70R 184V	0	1	0	1
74V 184V	0	0	2	2
184V 215Y	0	2	0	2
67N 184V 215F	1	0	0	1
70R 184V 219Q	0	1	0	1
74V 115F 184V	0	0	4	4
115F 184V 219E	0	0	1	1
41L 184V 210W 215Y	0	2	0	2
67N 70R 184V 219Q	1	1	0	2
70R 184V 215F 219Q	0	1	0	1
74V 115F 184V 219E	0	0	1	1
41L 67N 70R 184V 215F 219E 219Q	0	1	0	1
Number of TAMs				
0	17	13	15	45
1	0	3	2	5
2	1	1	0	2
3	1	4	0	5
6	0	1	0	1

Supplementary Figure 1 Monthly change in body circumferences and skinfold thicknesses from enrolment to week 96: differences between zidovudine vs stavudine and abacavir vs stavudine

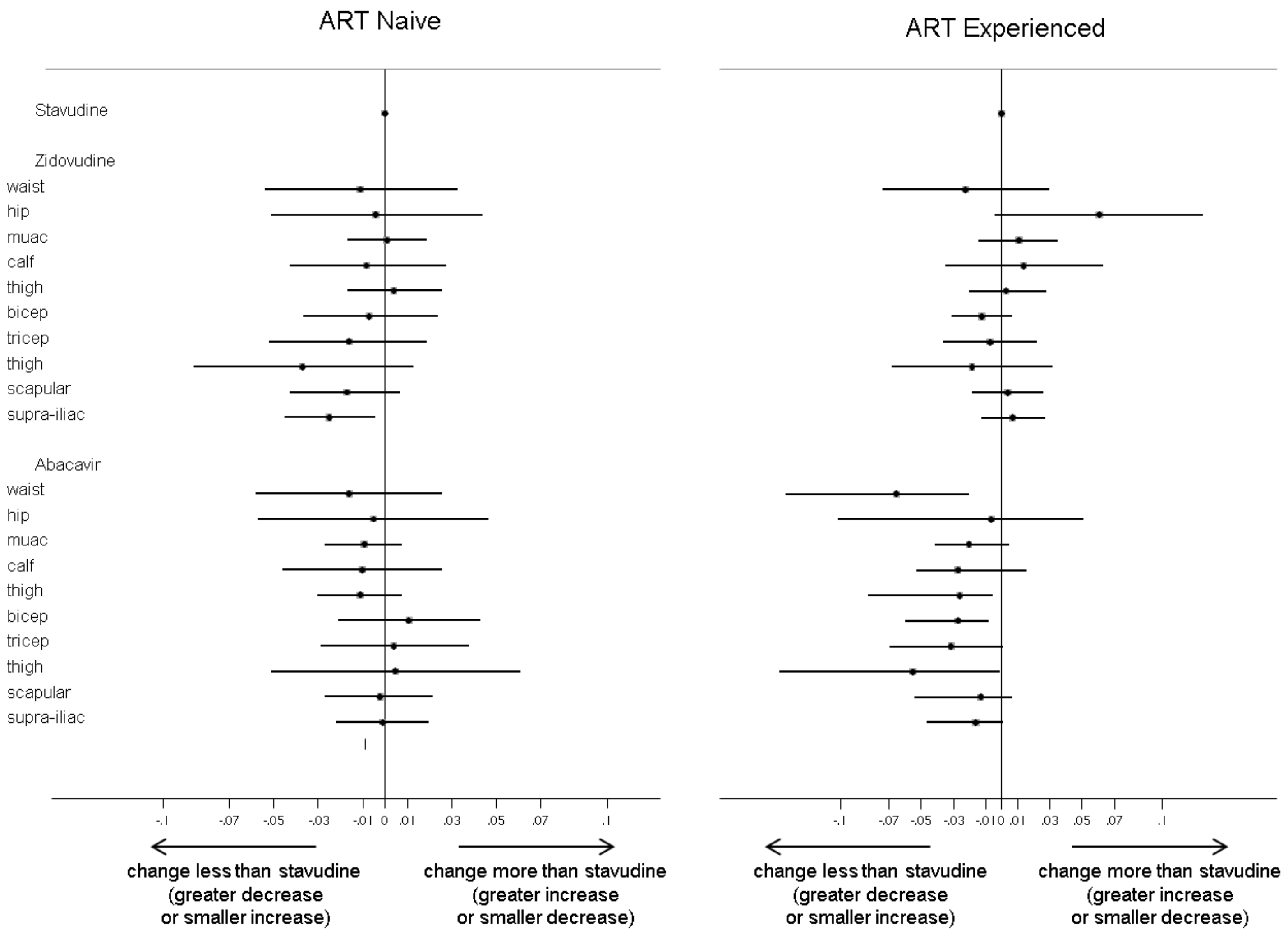

Supplementary Figure 2   CD4 response

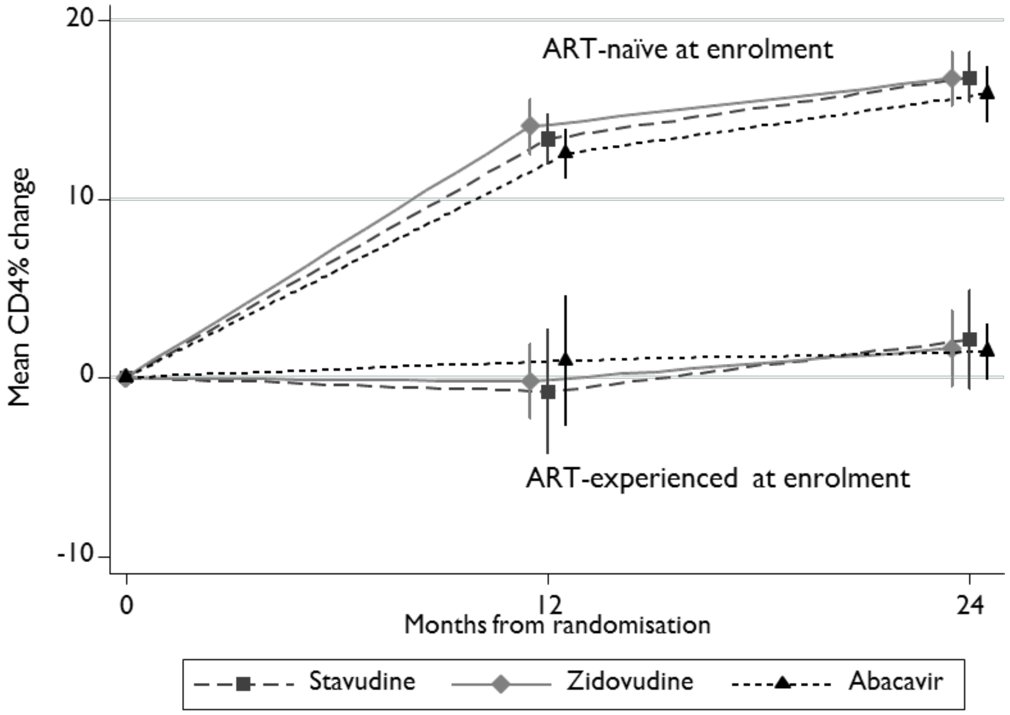

Supplementary Figure 3 Resistance at 96 weeks

3a, Major NRTI mutations in those >500 copies/ml at week 96

3b, Predicted intermediate/high level resistance in those >500 copies/ml at week 96

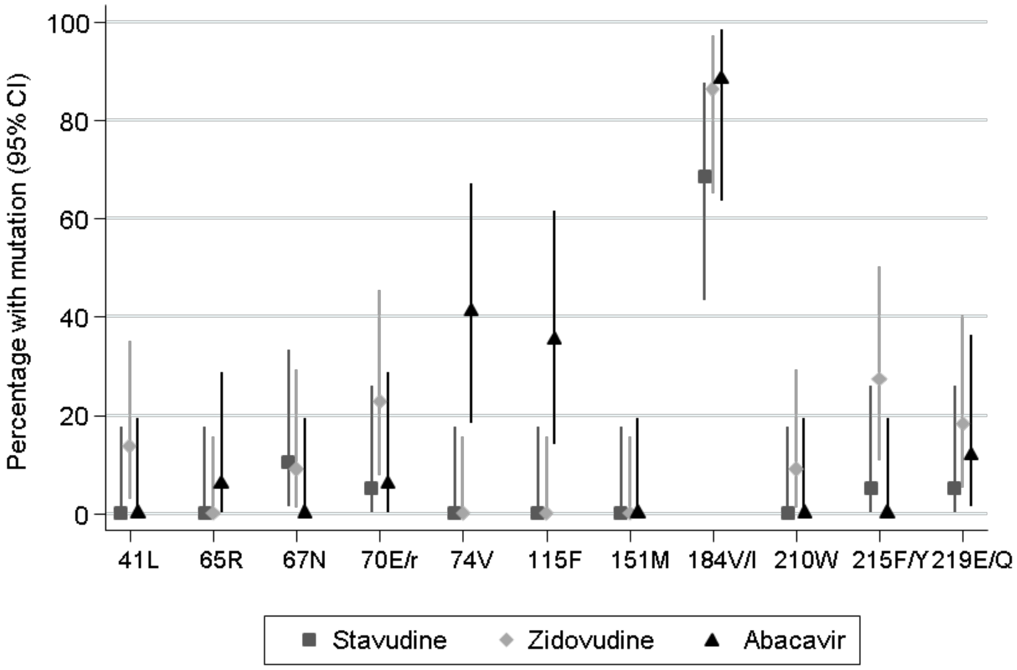

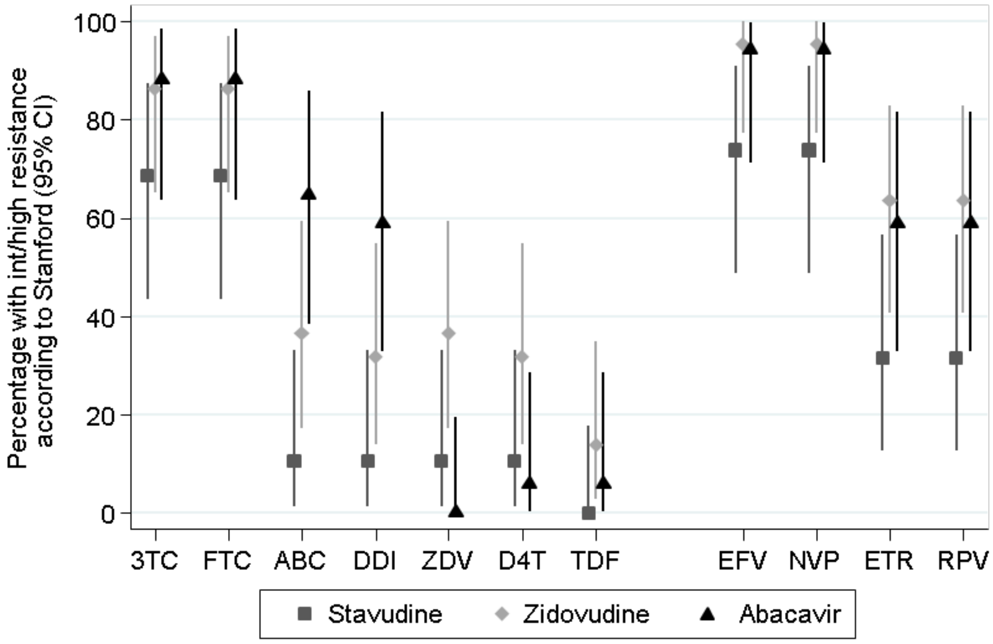

Note: int=intermediate, 3TC=lamivudine, FTC=emtricitabine, ABC=abacavir, DDI=didanosine, ZDV=zidovudine, D4T=stavudine, TDF=tenofovir, EFV=efavirenz, NVP=nevirapine, ETR=etravirine,RPV=rilpivirine.
